# Monitoring of gastrointestinal carcinoma via molecular residual disease with circulating tumor DNA using a tumor‐informed assay

**DOI:** 10.1002/cam4.6286

**Published:** 2023-08-21

**Authors:** Zining Qi, Yi Li, ZhengKun Wang, Xuerong Tan, Yixuan Zhou, Zhendong Li, Weirong Zhao, Xin Zheng, Jicheng Yao, Feng Li, Weifeng Wang, Zhizheng Wang, Fei Pang, Gang Wang, Weiguang Gu

**Affiliations:** ^1^ Department of Gastrointestinal surgery The First Hospital of Shanxi Medical University Taiyuan China; ^2^ Department of Gastrointestinal surgery The Affiliated Hospital of Qingdao University Qingdao China; ^3^ Department of Radiology The Second Hospital of Dalian Medical University Dalian China; ^4^ Shanghai OrigiMed Co., Ltd Shanghai China; ^5^ Department of General Surgery Jiangsu Province Hospital of Chinese Medicine Nanjing China; ^6^ Department of oncology Nanhai People's Hospital Foshan China; ^7^ Department of The Sixth Affiliated Hospital School of Medicine, South China University of Technology Foshan China

**Keywords:** circulating tumor DNA, gastrointestinal carcinoma, molecular residual disease, tumor metastasis

## Abstract

**Background:**

Circulating tumor DNA (ctDNA)‐based minimal residual disease (MRD) detection, which can identify disease relapse ahead of radiological imaging, has shown promising performance. The objective of this study was to develop and validate OriMIRACLE S (Minimal Residual Circulating Nucleic Acid Longitudinal Detection in Solid Tumor), a highly sensitive and specific tumor‐informed assay for MRD detection.

**Methods:**

Tumor‐specific somatic single nucleotide variants (SNVs) were identified via whole exome sequencing of tumor tissue and matched germline DNA. Clonal SNVs were selected using the OriSelector algorithm for patient‐specific, multiplex PCR‐based NGS assays in MRD detection. Plasma‐free DNA from patients with gastrointestinal tumors prior to and following an operation, and during monitoring, were ultradeep sequenced.

**Results:**

The detection of three positive sites was sufficient to achieve nearly 100% overall sample level sensitivity and specificity and was determined by calculating binomial probability based on customized panels containing 21 to 30 variants. A total of 127 patients with gastrointestinal tumors were enrolled in our study. Preoperatively, MRD was positive in 18 of 26 patients (69.23%). Following surgery, MRD was positive in 24 of 82 patients (29.27%). The positivity rate for MRD was 33.33% (*n* = 18) for gastric adenocarcinoma and 32.26% (*n* = 62) for colorectal cancer. Twenty (20) of 59 patients (34.48%) experienced a change in MRD status over the monitoring period. Patients 8 and 31 responded to 3 cycles of systemic therapy, after which levels for all ctDNA dropped below the detection limit. Patient 53 was an example of using MRD to predict tumor metastasis. Patient 55 showed a weak response to treatments first and respond to new systemic therapy after tumor progression.

**Conclusion:**

Our study identified a sensitive and specific clinical detection method for low frequency ctDNA, and explored the detection performance of this technology in gastrointestinal tumors.

## 1 INTRODUCTION

The evaluation of tumor disease status and the evaluation of treatment effect depend on radiation testing and serological testing.^1^ However, the results of radiological detection are vague and lack of sensitivity in clinical application, which is easy to confuse tumor with inflammatory reaction or scar. The detection of tumor serological markers also lacks sufficient sensitivity and specificity. Circulating tumor DNA‐based minimal residual disease (MRD) is an important biomarker in plasma of cancer patients, and is more and more widely used in clinic. The circulating tumor DNA (ctDNA) that can be detected in plasma has been proved to reflect the mutation characteristics of primary tumors, and is becoming a potential noninvasive biomarker for monitoring the progress of different cancer types. As a noninvasive method, ctDNA MRD sequencing is valuable in many clinical applications, such as early recurrence detection, tumor progression monitoring, and drug resistance mutation identification.^2^, ^3^ In many studies, the status of ctDNA MRD after surgery or systemic treatment are considered as high‐risk factors for recurrence.^4^, ^5^, ^6^ Monitoring the changes of ctDNA MRD before and after treatment can predict the therapeutic effect in the early stage of treatment.^7^, ^8^, ^9^

Reveal (Guardian health, Inc.) is a tumor agnostic analysis method that can simultaneously evaluate genomic mutations and methylation to detect residual disease and colorectal disease recurrence.^10^ In landmark plasma drawn one‐month postdefinitive therapy, 15 of 15 colorectal cancer patients had detectable ctDNA recurred and 12 of 49 CRC patients without detectable ctDNA recurred. Signatera (Natera) is a next‐generation ctDNA MRD detection and sequencing method based on individualization, tumor information, and multiplex polymerase chain reaction.^4^ ctDNA was detected in 108 (88.5%) of 122 preoperative blood samples of patients with stage I‐III colon cancer, and 10 (10.6%) of 94 plasma samples 30 days after operation were positive for ctDNA MRD. Seven of 10 patients had detectable ctDNA recurred and 10 of 84 CRC patients without detectable ctDNA recurred. In a study on colorectal cancer patients based on 15 gene panel sequencing, ctDNA was detected in 77%, 8.3%, and 12% of plasma samples before treatment, after radiotherapy and chemotherapy and after surgery, respectively.^11^ In advanced cancer, the maximal AF of ctDNA ranges from 0.1% to 10%, which is correlated with the cellular amount of tumor mass^12^ and tumor type.^13^ In a study of 12,337 cancer patients, Parallel sequencing of plasma and leukocytes based on the next‐generation sequencing method found that ctDNA MRD was detected in 73.5% of plasma samples, of which the detection rate of small‐cell lung cancer was the highest (91.1%) and thyroid cancer was the lowest (41.8%).^13^ In the early of cancer, ctDNA concentration is low.^3^, ^14^ More sensitive ctDNA monitoring technology is helpful to successfully detect low frequency ctDNA in plasma, and has more extensive clinical application value.

The objective of our study was to develop and validate a highly sensitive and specific tumor‐informed assay for ctDNA MRD detection via plasma cell‐free DNA. We tested the feasibility of plasma ctDNA for the noninvasive analysis of tumor mutations in gastrointestinal tumors by sequencing of tumor tissues, as well as presurgery and postsurgery plasma samples.

## 2 METHODS

### 2.1 Standard sample

Standard samples were developed as following for assessing assay performance. Somatic variant samples were consisted of a wild‐type sample as control and two commercialized standard samples each containing five positive sites at variant allele frequency [VAF] of 0.1% and 1%. Single nucleotide polymorphism (SNP) samples were made by blending three gDNA samples (160 sites) with gDNA samples without these SNPs to VAFs of 0%, 0.005%, 0.01%, 0.02%, 0.05%, and 0.1%. The cfDNA samples were prepared by titrating the clinical cfDNA mixture from five patients with 31 positive sites to 0.01%–0.3% and 0.1%–3% VAF with single nucleosome HEK293 DNA. The limit of detection (LOD), specificity, sensitivity, repeatability, and reproducibility of this assay were determined experimentally with a series of standard references and in silico.

### 2.2 Sample sensitivity and specificity

The intra assay accuracy rate was evaluated by the following method. The standard samples with horizon variation frequency of 1%, 0.1%, and wide type (WT) were selected, and each sample was repeated three times in one run. The inter batch accuracy was evaluated by the following method. The standard samples with Horizon variation frequency of 1%, 0.1%, and wild type were selected, and the experiment was repeated three times in three runs for each sample. For three pairs of WES samples, use the negative sample to dilute the paired positive sample. The gradient is 0.1%, 0.05%, 0.02%, 0.01%, and 0.005% in turn. Each gradient has three replicates. Three pairs of negative samples without SNP were amplified by additional primers with three replicates. According to SNP detection, the site sensitivity and specificity were calculated. Five ctDNA samples, one negative sample, and mixed SNV Mix (0.1%–3% and 0.01%–0.3%) were repeated three times for each case. According to the detection of SNV, the site sensitivity and specificity were counted. The SNV pool was set to 20–50 sites, and the SNV threshold was set to 2–5 sites. Simulate the SNP sites of mixed samples for 1000 times, judge positive and negative, and calculate the sensitivity at the sample level. Simulate the negative site for 1000 times, judge the negative and positive, and calculate the specificity of the sample level.

### 2.3 Patient and sample collection

Between 2021 and 2022, 127 patients with histologically confirmed gastric adenocarcinoma, colorectal cancer, or small intestine tumors in the first hospital of Shanxi medical university, the affiliated hospital of Qingdao university, the second hospital of Dalian medical university, Jiangsu province hospital of Chinese medicine and the sixth affiliated hospital south China University of technology were enrolled in our study. Tumors were obtained at the time of surgery. Peripheral blood samples were obtained the day prior to surgery or following surgery. For longitudinal ctDNA MRD monitoring, peripheral blood samples were obtained more than two times prior to or following surgery. The study was approved by the Ethics Committee of First Hospital of Shanxi Medical University (approval number 2022 K156) and the Ethics Committee of the Sixth Affiliated Hospital South China University of Technology (approval number 2022182). All participants provided written informed consent.

### 2.4 Sequencing and variant calling

Tumor‐specific somatic single nucleotide variants (SNVs) were identified via WES in tumor tissue and in matched germline DNA. SNV sites with clear clinical significance were selected, and primers were designed to form a patient personalized panel. More than 20 mutations per patient, with VAFs >10% in tumors, were prioritized for primer design.

### 2.5 MRD ctDNA detection

Cell‐free DNA (cfDNA) was isolated from plasma using the QIAamp Circulating Nuclear Acid Kit (QIAGEN, Venlo, Netherlands). Multiplex PCR was performed using a NEBNext Ultra II Q5 Master Mix (New England Biolabs, Inc., MA, USA). A library was constructed using the VAHTS® Universal DNA Library Prep Kit for Illumina V3 (Nanjing Vazyme Biotech Co. Ltd., Nanjing, China) and sequenced using a NovaSeq 6000 sequencer (Illumina Inc., CA, USA). For a SNP pool comprised of all mutations, two or more mutations were judged to be MRD positive, whereas one or no mutations were judged to be MRD negative.

### 2.6 Statistical analysis

The R Statistical Software package (version 3.4.3, R Foundation was used for Statistical Computing). The mean ± SD was used for continuous variable cfDNA content. *T*‐test was used for comparison between two groups. *p* value <0.05 was considered statistically significant.

## 3 RESULTS

### 3.1 Sensitivity and specificity

**FIGURE 1:**
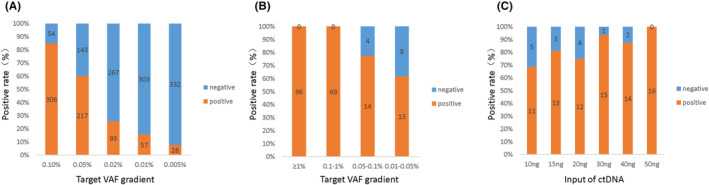
Mutation detection for different allele variation frequencies under different conditions. (A) Mutation detection for different allele variation frequencies in standard samples. (B) Mutation detection for different allele variation frequencies in blood samples. (C) Mutation detection for blood samples under different sample loading conditions.

Three pairs of WES samples were diluted with corresponding negative samples. The target VAF gradient was 0.1%, 0.05%, 0.02%, 0.01%, and 0.005%. To calculate the sensitivity of sites at different frequencies, each gradient was detected three times (Figure 1A). Three pairs of negative samples without SNP were detected using the same method and repeated three times. Five false positive sites were detected in 462 negative sites in three negative samples, indicating that the specificity is 98.9% (Table S1). Once five ctDNA samples and one negative sample were mixed, the VAF of the mixed mutation was 0.1%–3% and 0.01%–0.3%. To calculate the sensitivity of sites at different frequencies, each sample was detected three times (Figure 1B). Seventy‐eight (78) sites of negative samples were detected three times, corresponding to five ctDNA positive samples. Test results were all negative, and ctDNA site specificity was 100%.

A library was constructed using a SNV mix (0.01–0.3%) of ctDNA diluted samples with different input starting concentrations (10 ng, 15 ng, 20 ng, 30 ng, 40 ng, and 50 ng), and was used to calculate the positive rate of site detection under different sample loading quantities (Figure 1C). Each SNV poll was set using 20–50 loci, and the positive threshold was set using 2–5 SNV. To determine whether or not a sample was negative or positive and to calculate sample level sensitivity, 1000 simulations on SNP sites of mixed samples were performed (Table 1).

**TABLE 1:** Sensitivity and specificity of samples under different SNP pool number and SNV threshold conditions.

SNP pool	20	20	20	20	22	22	22	22
SNV threshold	2	3	4	5	2	3	4	5
Sample sensitivity	100.0%	99.4%	97.3%	93.0%	100.0%	100.0%	98.6%	96.4%
Sample specificity	98.9%	99.9%	100.0%	100.0%	97.7%	99.9%	100.0%	100.0%
SNP pool	24	24	24	24	26	26	26	26
SNV threshold	2	3	4	5	2	3	4	5
Sample sensitivity	100.0%	99.7%	99.4%	98.0%	100.0%	99.9%	99.6%	99.2%
Sample specificity	97.1%	99.8%	100.0%	100.0%	97.0%	99.8%	100.0%	100.0%
SNP pool	28	28	28	28	30	30	30	30
SNV threshold	2	3	4	5	2	3	4	5
Sample sensitivity	100.0%	100.0%	100.0%	99.5%	100.0%	100.0%	100.0%	99.9%
Sample specificity	97.1%	99.8%	100.0%	100.0%	96.6%	99.6%	99.9%	100.0%
SNP pool	32	32	32	32	34	34	34	34
SNV threshold	2	3	4	5	2	3	4	5
Sample sensitivity	100.0%	100.0%	100.0%	100.0%	100.0%	100.0%	100.0%	99.8%
Sample specificity	96.1%	99.3%	99.9%	100.0%	95.8%	99.3%	100.0%	100.0%
SNP pool	36	36	36	36	38	38	38	38
SNV threshold	2	3	4	5	2	3	4	5
Sample sensitivity	100.0%	100.0%	100.0%	100.0%	100.0%	100.0%	100.0%	100.0%
Sample specificity	94.4%	99.5%	100.0%	100.0%	95.2%	99.3%	100.0%	100.0%
SNP pool	40	40	40	40	42	42	42	42
SNV threshold	2	3	4	5	2	3	4	5
Sample sensitivity	100.0%	100.0%	100.0%	100.0%	100.0%	100.0%	100.0%	100.0%
Sample specificity	93.1%	99.5%	100.0%	100.0%	93.7%	99.1%	99.7%	100.0%
SNP pool	44	44	44	44	46	46	46	46
SNV threshold	2	3	4	5	2	3	4	5
Sample sensitivity	100.0%	100.0%	100.0%	100.0%	100.0%	100.0%	100.0%	100.0%
Sample specificity	93.2%	98.9%	99.8%	100.0%	94.1%	99.4%	100.0%	99.9%
SNP pool	48	48	48	48	50	50	50	50
SNV threshold	2	3	4	5	2	3	4	5
Sample sensitivity	100.0%	100.0%	100.0%	100.0%	100.0%	100.0%	100.0%	100.0%
Sample specificity	91.5%	99.2%	99.9%	100.0%	91.2%	98.3%	99.8%	100.0%

### 3.2 Patient characteristics

**FIGURE 2:**
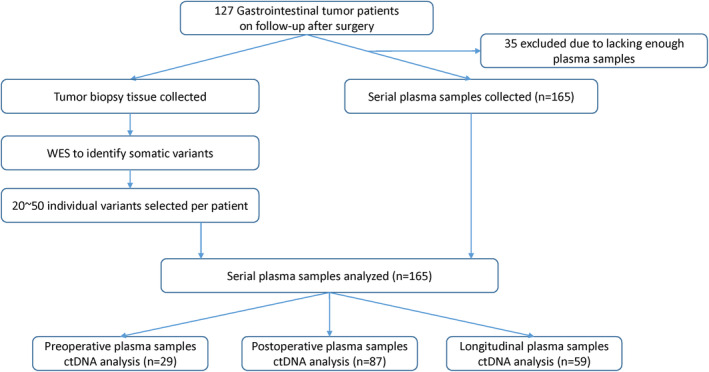
Patient enrollment, sample collection, and definitions for patient subgroups. For 127 patients with gastrointestinal tumor in this study, exonic alterations were determined through paired‐end sequencing of formalin‐fixed, paraffin‐embedded tumor tissue specimens, and matched normal DNA. Patient‐specific panels were designed to include more than 20 somatic mutations identified from WES data. A total of 29 preoperative plasma samples and 87 postoperative plasma samples were analyzed for ctDNA detection. A total of 165 plasma samples from 92 patients were analyzed for ctDNA detection.

A total of 127 patients with gastrointestinal tumors were enrolled for ctDNA MRD detection, including 27 gastric, 92 colorectal, and eight small intestine tumors (Figure 2). Among patients evaluated, 35 were excluded due to a lack of blood samples. The median age of the remaining 92 patients at the time of diagnosis was 60 years old (range 29–79), and 54.35% (*n* = 50) were men (Table 2). Seventy percent patients received radical resection, 17% patients received local resection, and 29% patients received systematic treatment including FOFLOX, XELOX, bevacizumab, etc. Patient characteristics and demographics are provided in the Supplementary materials.

**TABLE 2:** Patient demographics and baseline characteristics.

Patient characteristic	All patients (*n* = 92)
Age——median	60 (29–79)
Sex	
Male	50
Female	42
Tumor type	
Gastric adenocarcinoma	18
Colorectal adenocarcinoma	67
Small intestinal tumor	7
Differentiation grade	
Low differentiation	21
Middle low differentiation	10
Middle differentiation	53
Middle high differentiation	3
High differentiation	1
Unknown	4
Tumor stage	
I	11
II	15
III	32
IV	24
Unknown	10
Operation type	
Radical operation	65
Local resection of tumor	16
Other	11
Lymphatic node metastasis	
Yes	53
None	30
Unknown	9
Vascular invasion	
Yes	28
None	51
Unknown	13
Perineural invasion	
Yes	26
None	53
Unknown	13
Surgical margin	
Positive	1
Negative	78
Unknown	13
Microsatellite instability state	
MSS	85
MSI‐H	7

### 3.3 Preoperative detection of ctDNA MRD

**FIGURE 3:**
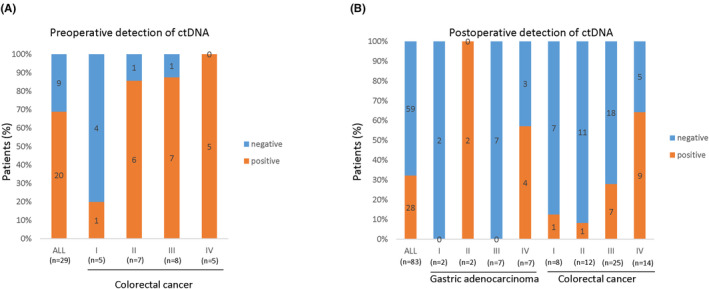
The preoperative (A) and postoperative (B) detection of ctDNA in gastrointestinal tumor patients. Preoperative plasma samples were taken at the time of surgery or the day before surgery. Postoperative blood samples were taken from 1 day to 134 days after operation, and the median sampling time was 31 days after operation.

Whole exome sequencing of tumors and matched germline DNA was used to identify somatic mutations. Tumor‐specific multiplex PCR assay panels that targeted 20–50 mutations were designed for each patient. The Somatic mutation and monitoring sites of each patient are provided in the Appendix S1. Ultradeep multiplex PCR–based NGS (median target coverage >105,000 reads) was used to analyze and quantify ctDNA in 165 plasma samples from 92 patients (Figure 2).In the 29 baseline preoperative plasma samples, ctDNA MRD was detected in 20 of 29 samples (68.97%), with a positivity rate of 20% for Stage I colorectal cancer (*n* = 5), 85.71% for Stage II colorectal cancer (*n* = 7), 87.5% for Stage III colorectal cancer (*n* = 8), and 100% for Stage IV colorectal cancer (*n* = 5), Figure 3A.

### 3.4 Postoperative detection of ctDNA MRD

In the 87 postoperative plasma samples, ctDNA MRD was detected in 28 of 87 samples (32.18%), with a positivity rate of 33.33% for gastric adenocarcinoma (*n* = 18), 0% for small intestine tumors (*n* = 6), and 33.33% for colorectal cancer (*n* = 63), Figure 3B. In more detail, the positivity rate was 57.14% for Stage IV gastric adenocarcinoma (*n* = 7), 12.5% for Stage I colorectal cancer (*n* = 8), 8.33% for Stage II colorectal cancer (*n* = 12), 28% for Stage III colorectal cancer (*n* = 25), and 64.29% for Stage IV colorectal cancer (*n* = 14), Figure 3B.

### 3.5 Longitudinal ctDNA MRD monitoring

**FIGURE 4:**
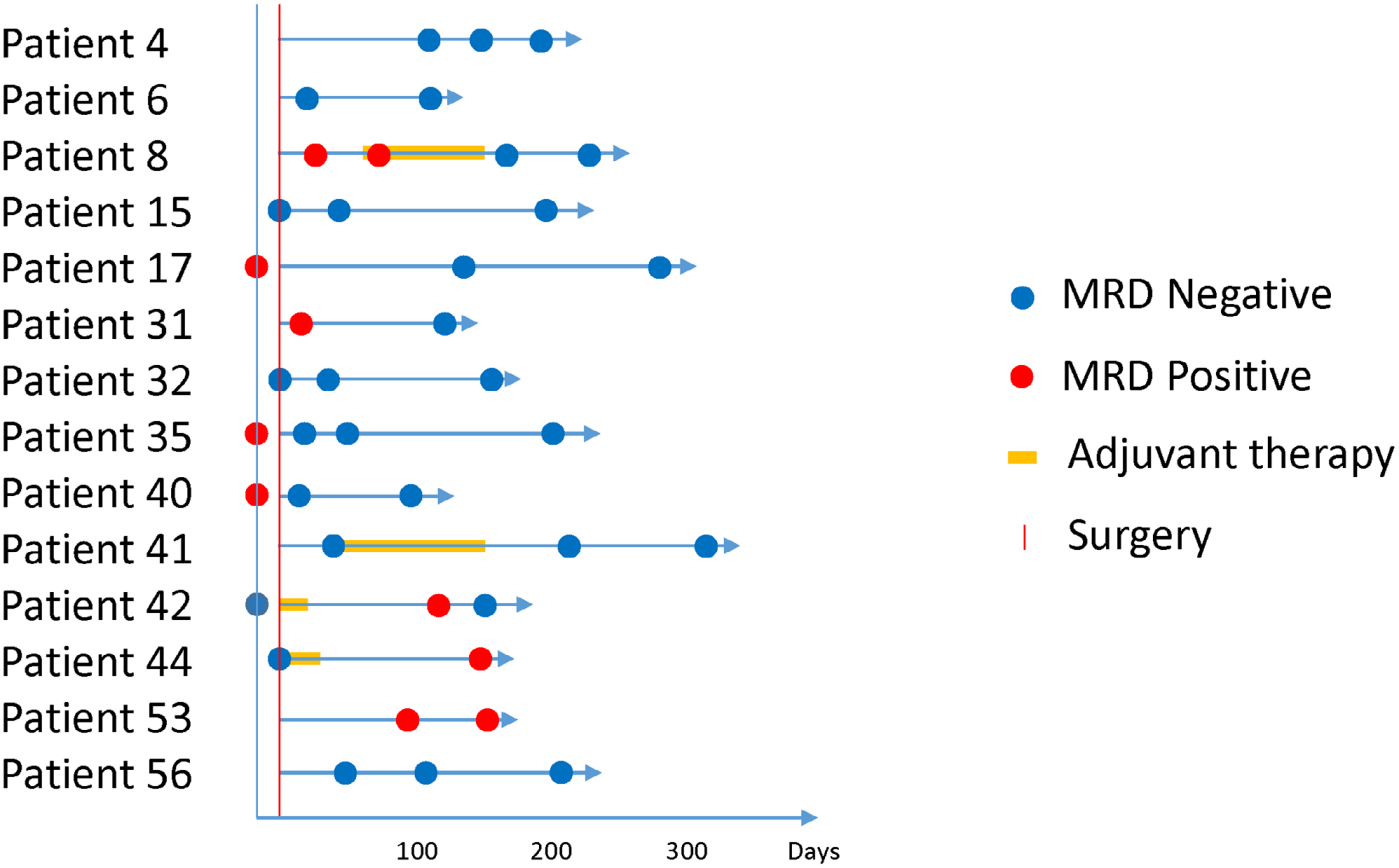
The longitudinal monitoring of serial plasma samples. The longitudinal monitoring of serial plasma samples in 14 patients, indicating when ctDNA was detected (red circle) or not detected (blue circle).

Fifty‐nine (59) patients had two or more blood samples taken at intervals, of which 20 patients experienced negative to positive or positive to negative changes (Table S2). Sixteen (16) patients tested positive for MRD prior to their operation, and 11 of these patients were negative following their operation. These results indicate that ctDNA in blood was significantly reduced following an operation. To categorize longitudinal data, the ctDNA level for each time point was expressed as a binary format (i.e., ctDNA MRD positive and negative) using a swimmer plot (Figure 4). Among the 35 cases for which ctDNA MRD was positive in a longitudinal manner, five (5) cases displayed evaluable ctDNA dynamics in response to treatment, whereas 24 cases displayed ctDNA MRD negative status over an extended period of time. As such, the results confirmed a post‐treatment, relapse‐free status.

In order to find out whether the MRD test result is caused by the change of blood cfDNA content before and after the operation, the difference of the total amount of cfDNA contained in 10 mL blood samples before and after the operation was compared (Figure S1). The average cfDNA content before the operation was 6.948 ng/mL, and there was no significant difference between the average cfDNA content of 7.764 ng/mL, 7.321 ng/mL, and 6.72 ng/mL of multiple blood samples after the operation.

### 3.6 ctDNA for monitoring the efficacy of systemic therapy

**FIGURE 5:**
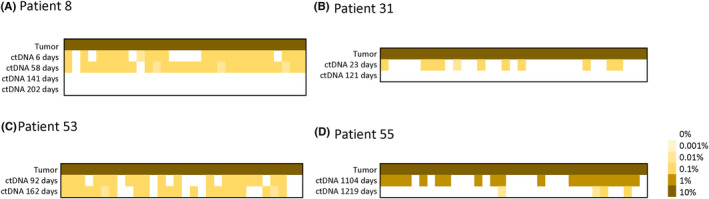
Examples of the longitudinal monitoring of ctDNA in four patients. The heatmap shows signals from different variants. Each column represents a different variant and each row represents a different sample type.

Patient 8 was diagnosed with rectal ulcer type moderately differentiated tubular adenocarcinoma, with a tumor size of 3.5 × 3.5 cm, which invaded the muscular layer and the fibrous adipose tissue outside the muscular layer, and had nerve invasion. Following 3D laparoscopic assisted radical resection of rectal cancer under general anesthesia, 198 individual somatic mutations were detected via WES in tumor tissue. Thirty (30) sites were used to design a monitoring panel. ctDNA MRD positive status was detected on the sixth day after operation (Figure 5A). Twenty‐two (22) sites were detected, with an average VAF of 0.143%. One month later, a CT scan indicated that there may be a metastatic tumor in S8 of the liver. The patient then began to receive cetuximab, oxaliplatin, and raltitrexed treatment. At the beginning of treatment, ctDNA MRD sample monitoring indicated that 28 sites could be monitored, with an average VAF of 0.096%. During treatment, two rounds of CT scans indicated that liver metastasis was smaller than before, suggesting that the treatment was effective. Following 3 cycles of treatment, ctDNA MRD monitoring was conducted and all sites were negative. ctDNA MRD was once again determined negative after 2 months.

Patient 31, a 70‐year‐old male, presented with melena, abdominal pain, and abdominal distension. A colonoscopy revealed a sigmoid colon tumor. Subsequently, a laparoscopic sigmoidectomy was performed under general anesthesia. The postoperative pathology was moderately differentiated adenocarcinoma, pT3N1a stage. One‐hundred and thirty‐four (134) individual cell mutations were detected via a WES in tumor tissue. KRAS exon 2 mutations were also detected. ctDNA MRD sample monitoring indicated that 11 sites were detected with an average VAF of 0.118% (Figure 5C). After three courses of chemotherapy with oxaliplatin and capecitabine, MRD detection was negative. Patient 55 was diagnosed as gastric adenocarcinoma and then underwent radical surgery for gastric cancer. The tumor size was 2 × 2 × 0.8 cm, Stage IIIB. Following surgery, the patient received eight treatments of an intraperitoneal infusion of docetaxel and tegafur. After 3 years, CA 19–9 increased to 375.6 u/mL, and ctDNA MRD was positive (Figure 5D), with an average VAF of 4.45%. However, no recurrence or metastasis was determined via imaging. The patient requested active treatment and received 2 cycles of treatment with apatinib combined with a PD‐1 monoclonal antibody. One month later, CA19‐9 continued to rise to 668.1 u/mL, and a CT scan indicated abnormal enhancement in the liver. The patient then received oxaliplatin plus tegafur plus cisplatin. After 3 months, ctDNA MRD was positive, but the average VAF decreased to 0.06%. Meanwhile, CEA gradually decreased to 152.6 u/mL.

### 3.7 CtDNA for monitoring recurrence or metastasis

Patient 53, with left upper abdominal pain for more than 1 month, was diagnosed, via gastroscopy, with a gastric malignant tumor. A laparoscopic proximal gastrectomy was then performed. The patient was pathologically diagnosed with moderately poorly differentiated adenocarcinoma at the gastroesophageal junction, ulcerative type, 10 × 4 × 2 cm in size. According to the Laurens' classification, the type was mixed, and invaded the serosa. Using WES, 164 individual somatic mutations were detected in tumor tissue. Thirty (30) sites were used to design a monitoring panel. A ctDNA MRD positive determination was found 3 months later (Figure 5B). Nineteen (19) sites were detected, with an average VAF of 0.123%. Three months later, a MRD monitoring was again performed, 21 sites were detected, and the average VAF was 0.138%. Subsequently, a CT scan indicated multiple metastases in the liver, and 1 month later a CT scan indicated that the metastases were larger than before.

## 4 DISCUSSION

At present, there are many methods for detecting ctDNA MRD in blood, such as single or multiple locus detection based on PCR,^6^, ^11^, ^15^, ^16^, ^17^, ^18^ gene panel sequencing based on second‐generation sequencing,^19^, ^20^, ^21^, ^22^ whole exome detection based on second‐generation sequencing, and multiplex PCR detection.^4^, ^23^ Among them, PCR‐based site detection is fast and economical, but it needs to obtain tumor related mutations in advance and cannot discover the evolution and changes of tumor cells. Gene panel sequencing based on second‐generation sequencing can theoretically include high frequency genes to cover most patients, and can detect the evolution and change of tumors to a certain extent. However, it is necessary to select the appropriate genes to design the panel for different tumors, and requires a larger sequencing range and higher cost. ctDNA of all cancer types can be detected by whole exome sequencing of tumor tissues followed by personalized targeted multiplex sequencing of plasma.

The sensitivity of single site detection will decrease significantly with the decrease of target gene VAF. Increasing the sample loading amount can improve the detection positive rate to a certain extent, and increasing the number of detection sites can greatly improve the sensitivity of samples. However, as the number of detection sites increases, the false positive probability of samples increases and the specificity of sample detection decreases. Increasing the SNV threshold can improve the specificity, but it will reduce the detection sensitivity. Therefore, setting an appropriate SNV threshold according to the number of detection sites can control both sensitivity and specificity at a high level. Multiplex PCR detects 20–50 variant sites simultaneously, achieving a very low detection limit at low cost, and overcoming intratumoral heterogeneity to a certain extent.

In the preoperative situation, the detection rate was high in patients with advanced stage and relatively low in patients with stage I. The detection rate was similar to that of previous studies,^4^, ^10^ which verified our ctDNA MRD detection technology. We also collected plasma samples after surgery. Compared with plasma samples before surgery, the detection rate of ctDNA MRD after surgery was significantly reduced, suggesting that ctDNA MRD is a valuable biomarker to evaluate tumor status and treatment effect. We also monitored the change of ctDNA MRD throughout the disease process by longitudinal ctDNA MRD analysis of patients. The reliability of this technique was further supported by comparing the changes of ctDNA MRD and clinical conditions of patients.

In preoperative blood samples, the detection rate of ctDNA in patients with stage II‐IV colorectal cancer was 90%, while that in patients with stage I was 20% lower. In postoperative blood samples, 30.51% of colorectal cancer patients detected ctDNA. Based on the detection technology of ctDNA mutation or methylation by digital PCR, 63 of 82 (76.8%) patients with metastatic colorectal cancer detected ctDNA at baseline.^17^ Based on the Safe SeqS test, ctDNA was detectable in 77%, 8.3%, and 12% of prevention, postmoradiotherapy, and postsurgery in patients with locally advanced rectal cancer.^11^ Based on the 425 gene panel sequenced in the second generation, ctDNA was detected in 154 of 240 colorectal cancer patients' preoperative blood samples, with 65.2% in stage II CRC and 63.3% in stage III colon cancer, while the positive rate of ctDNA after surgery decreased to 8.3%.^19^ In the netare study, 108 of 122 (88.5%) colorectal cancer patients detected ctDNA before surgery, including 40% in stage I, 92% in stage II, and 90% in stage III. The positive rate of ctDNA after surgery decreased to 10.6%.^4^ We also collected plasma samples after surgery. Compared with the preoperative plasma samples, the detection rate of ctDNA MRD after surgery was significantly reduced, which indicates that ctDNA MRT is a valuable biomarker for evaluating tumor status and therapeutic effect. We also monitored the changes of ctDNA MRD during the whole disease process by longitudinal ctDNA MRD analysis of patients. By comparing the changes of ctDNA MRD and the clinical conditions of patients, the reliability of this technique is further supported. At present, there are only a few studies on ctDNA MRD of gastric cancer.^24^, ^25^, ^26^ ctDNA was positive in preoperative blood samples 20 of 49 stage I‐III gastric cancer and seven of 38 postoperative blood samples.^25^ ctDNA was positive in preoperative blood samples 11 of 19 advanced gastric cancer patients and eight of 19 postoperative blood samples.^24^ The detection rate of plasma ctDNA MRD in patients with gastric cancer after operation was similar to that of patients with intestinal cancer. Longitudinal ctDNA MRD analysis also found that the changes of plasma ctDNA MRD in patients with gastric cancer were closely related to the patient's condition. These results indicate that this technique can overcome the heterogeneity of tumor variation among different cancer species, and can be applied not only to colorectal cancer but also to gastric cancer patients.

Our study preliminarily explored the possibility of using this technology to monitor patients' disease changes in patients with gastrointestinal cancer. When using this technology for disease monitoring, it is necessary to comprehensively judge the changes of patients' tumors based on cancer type, number of tumor gene mutations, number of available and monitored gene mutations, and treatment methods. Like all technologies, the method outlined has certain limitations. First of all, it is necessary to have enough ctDNA molecules in the collected plasma, and the ability of different tumors to release ctDNA to plasma species may be different.^13^ In this study, the positive rate of ctDNA MRD in the blood of patients with small bowel cancer is significantly lower than that of gastric cancer or colorectal cancer, which may be related to this factor. Secondly, under the influence of tumor heterogeneity and drug selection, the monitoring points of recurrent tumors may disappear, thus affecting the application of technology in the whole tumor monitoring process. Finally, the number of samples collected in this study is limited, and the ctDNA MRD positive rate under different stages may change with the increase of the sample size. More samples need to be collected to obtain more reliable conclusions.

In conclusion, in this study, we propose a sensitive and specific method for clinical detection of low frequency ctDNA, and explored the sensitivity and specificity under different detection sites and different thresholds condition. The performance of this technology was verified in colorectal cancer patients, and the feasibility of this technology in gastric cancer was explored. This method can be used in the future to identify patients with high‐risk recurrent gastrointestinal tumors and monitor their response to systematic treatment.

## AUTHOR CONTRIBUTIONS

**Zining Qi:** Data curation (equal); investigation (equal); resources (equal); supervision (equal); validation (equal); writing – original draft (equal); writing – review and editing (equal). **Yi Li:** Data curation (equal); investigation (equal); resources (equal); writing – original draft (equal). **ZhengKun Wang:** Data curation (equal); investigation (equal); resources (equal). **Xuerong Tan:** Data curation (equal); investigation (equal); resources (equal). **Yixuan Zhou:** Data curation (equal); investigation (equal); resources (equal). **Zhendong Li:** Methodology (equal); software (equal). **Weirong Zhao:** Methodology (equal); software (equal). **Xin Zheng:** Methodology (equal); software (equal). **Jicheng Yao:** Methodology (equal); software (equal). **Feng Li:** Methodology (equal); software (equal). **Weifeng Wang:** Methodology (equal); software (equal). **Zhizheng Wang:** Data curation (equal); writing – original draft (equal). **Fei Pang:** Project administration (equal); supervision (equal). **Gang Wang:** Project administration (equal); resources (equal); supervision (equal); writing – review and editing (equal). **Weiguang Gu:** Project administration (equal); resources (equal); supervision (equal); writing – review and editing (equal).

## Supporting information


Appendix S1

Figure S1

Tables S1–S2

## Data Availability

The data that support the findings of this study are available from the corresponding author upon reasonable request.

## References

[1] Shah M , Denlinger CS . Optimal post‐treatment surveillance in cancer survivors: is more really better? Oncology (Williston Park). 2015;29(4):230‐240. 25952485

[2] Bettegowda C , Sausen M , Leary RJ , et al. Detection of circulating tumor DNA in early‐ and late‐stage human malignancies. Sci Transl Med. 2014;6(224):224ra24. 10.1126/scitranslmed.3007094 PMC4017867 24553385

[3] Russo M , Siravegna G , Blaszkowsky LS , et al. Tumor heterogeneity and lesion‐specific response to targeted therapy in colorectal cancer. Cancer Discov. 2016;6(2):147‐153. 26644315 10.1158/2159-8290.CD-15-1283 PMC4744519

[4] Reinert T , Henriksen TV , Christensen E , et al. Analysis of plasma cell‐free DNA by Ultradeep sequencing in patients with stages I to III colorectal cancer. JAMA Oncol. 2019;5(8):1124‐1131. 31070691 10.1001/jamaoncol.2019.0528 PMC6512280

[5] Wang Y , Li L , Cohen JD , et al. Prognostic potential of circulating tumor DNA measurement in postoperative surveillance of nonmetastatic colorectal cancer. JAMA Oncologia. 2019;5(8):1118‐1123. 10.1001/jamaoncol.2019.0512 PMC6512291 31070668

[6] Tie J , Cohen JD , Wang Y , et al. Circulating tumor DNA analyses as markers of recurrence risk and benefit of adjuvant therapy for stage III colon cancer. JAMA Oncol. 2019;5(12):1710‐1717. 31621801 10.1001/jamaoncol.2019.3616 PMC6802034

[7] Henriksen TV , Tarazona N , Frydendahl A , et al. Circulating tumor DNA in stage III colorectal cancer, beyond minimal residual disease detection, toward assessment of adjuvant therapy efficacy and clinical behavior of recurrences. Clin Cancer Res. 2022;28(3):507‐517. 34625408 10.1158/1078-0432.CCR-21-2404 PMC9401484

[8] Taieb J , Taly V , Henriques J , et al. Prognostic value and relation with adjuvant treatment duration of ctDNA in stage III colon cancer: a post hoc analysis of the PRODIGE‐GERCOR IDEA‐France trial. Clin Cancer Res. 2021;27(20):5638‐5646. 34083233 10.1158/1078-0432.CCR-21-0271

[9] Tie J , Cohen JD , Lahouel K , et al. Circulating tumor DNA analysis guiding adjuvant therapy in stage II colon cancer. N Engl J Med. 2022;386(24):2261‐2272. 35657320 10.1056/NEJMoa2200075 PMC9701133

[10] Parikh AR , Van Seventer EE , Siravegna G , et al. Minimal residual disease detection using a plasma‐only circulating tumor DNA assay in patients with colorectal cancer. Clin Cancer Res. 2021;27(20):5586‐5594. 33926918 10.1158/1078-0432.CCR-21-0410 PMC8530842

[11] Tie J , Cohen JD , Wang Y , et al. Serial circulating tumour DNA analysis during multimodality treatment of locally advanced rectal cancer: a prospective biomarker study. Gut. 2019;68(4):663‐671. 29420226 10.1136/gutjnl-2017-315852 PMC6265124

[12] Abbosh C , Birkbak NJ , Wilson GA , et al. Phylogenetic ctDNA analysis depicts early‐stage lung cancer evolution. Nature. 2017;545(7655):446‐451. 28445469 10.1038/nature22364 PMC5812436

[13] Zhang Y , Yao Y , Xu Y , et al. Pan‐cancer circulating tumor DNA detection in over 10,000 Chinese patients. Nat Commun. 2021;12(1):11. 33397889 10.1038/s41467-020-20162-8 PMC7782482

[14] Diehl F , Schmidt K , Choti MA , et al. Circulating mutant DNA to assess tumor dynamics. Nat Med. 2008;14(9):985‐990. 18670422 10.1038/nm.1789 PMC2820391

[15] Tie J , Kinde I , Wang Y , et al. Circulating tumor DNA as an early marker of therapeutic response in patients with metastatic colorectal cancer. Ann Oncol. 2015;26(8):1715‐1722. 25851626 10.1093/annonc/mdv177 PMC4511218

[16] Tie J , Wang Y , Tomasetti C , et al. Circulating tumor DNA analysis detects minimal residual disease and predicts recurrence in patients with stage II colon cancer. Sci Transl Med. 2016;8(346):346ra92. 10.1126/scitranslmed.aaf6219 PMC5346159 27384348

[17] Garlan F , Laurent‐Puig P , Sefrioui D , et al. Early Evaluation of circulating tumor DNA as marker of therapeutic efficacy in metastatic colorectal cancer patients (PLACOL study). Clin Cancer Res. 2017;23(18):5416‐5425. 28576867 10.1158/1078-0432.CCR-16-3155

[18] Tarazona N , Gimeno‐Valiente F , Gambardella V , et al. Targeted next‐generation sequencing of circulating‐tumor DNA for tracking minimal residual disease in localized colon cancer. Ann Oncol. 2019;30(11):1804‐1812. 31562764 10.1093/annonc/mdz390

[19] Chen G , Peng J , Xiao Q , et al. Postoperative circulating tumor DNA as markers of recurrence risk in stages II to III colorectal cancer. J Hematol Oncol. 2021;14(1):80. 34001194 10.1186/s13045-021-01089-z PMC8130394

[20] Wang DS , Yang H , Liu XY , et al. Dynamic monitoring of circulating tumor DNA to predict prognosis and efficacy of adjuvant chemotherapy after resection of colorectal liver metastases. Theranostics. 2021;11(14):7018‐7028. 34093868 10.7150/thno.59644 PMC8171084

[21] Wang Y , Yang L , Bao H , et al. Utility of ctDNA in predicting response to neoadjuvant chemoradiotherapy and prognosis assessment in locally advanced rectal cancer: a prospective cohort study. PLoS Med. 2021;18(8):e1003741. 34464382 10.1371/journal.pmed.1003741 PMC8407540

[22] Li Y , Mo S , Zhang L , et al. Postoperative circulating tumor DNA combined with consensus molecular subtypes can better predict outcomes in stage III colon cancers: a prospective cohort study. Eur J Cancer. 2022;169:198‐209. 35636041 10.1016/j.ejca.2022.04.010

[23] Loupakis F , Sharma S , Derouazi M , et al. Detection of molecular residual disease using personalized circulating tumor DNA assay in patients with colorectal cancer undergoing resection of metastases. JCO Precis Oncol. 2021;5:PO.21.00101. 34327297 10.1200/PO.21.00101 PMC8315303

[24] Kim YW , Kim YH , Song Y , et al. Monitoring circulating tumor DNA by analyzing personalized cancer‐specific rearrangements to detect recurrence in gastric cancer. Exp Mol Med. 2019;51(8):1‐10. 10.1038/s12276-019-0292-5 PMC6802636 31395853

[25] Yang J , Gong Y , Lam VK , et al. Deep sequencing of circulating tumor DNA detects molecular residual disease and predicts recurrence in gastric cancer. Cell Death Dis. 2020;11(5):346. 32393783 10.1038/s41419-020-2531-z PMC7214415

[26] van Velzen MJM , Creemers A , van den Ende T , et al. Circulating tumor DNA predicts outcome in metastatic gastroesophageal cancer. Gastric Cancer. 2022;25(5):906‐915. 35763187 10.1007/s10120-022-01313-w PMC9365750

