# Supplementary figures and images for: Monitoring of gastrointestinal carcinoma via molecular residual disease with circulating tumor DNA using a tumor‐informed assay
Source: Cancer Med. 2023 Aug 21;12(16):16687–96. doi: 10.1002/cam4.6286 (PMC10501225; doi:10.1002/cam4.6286)

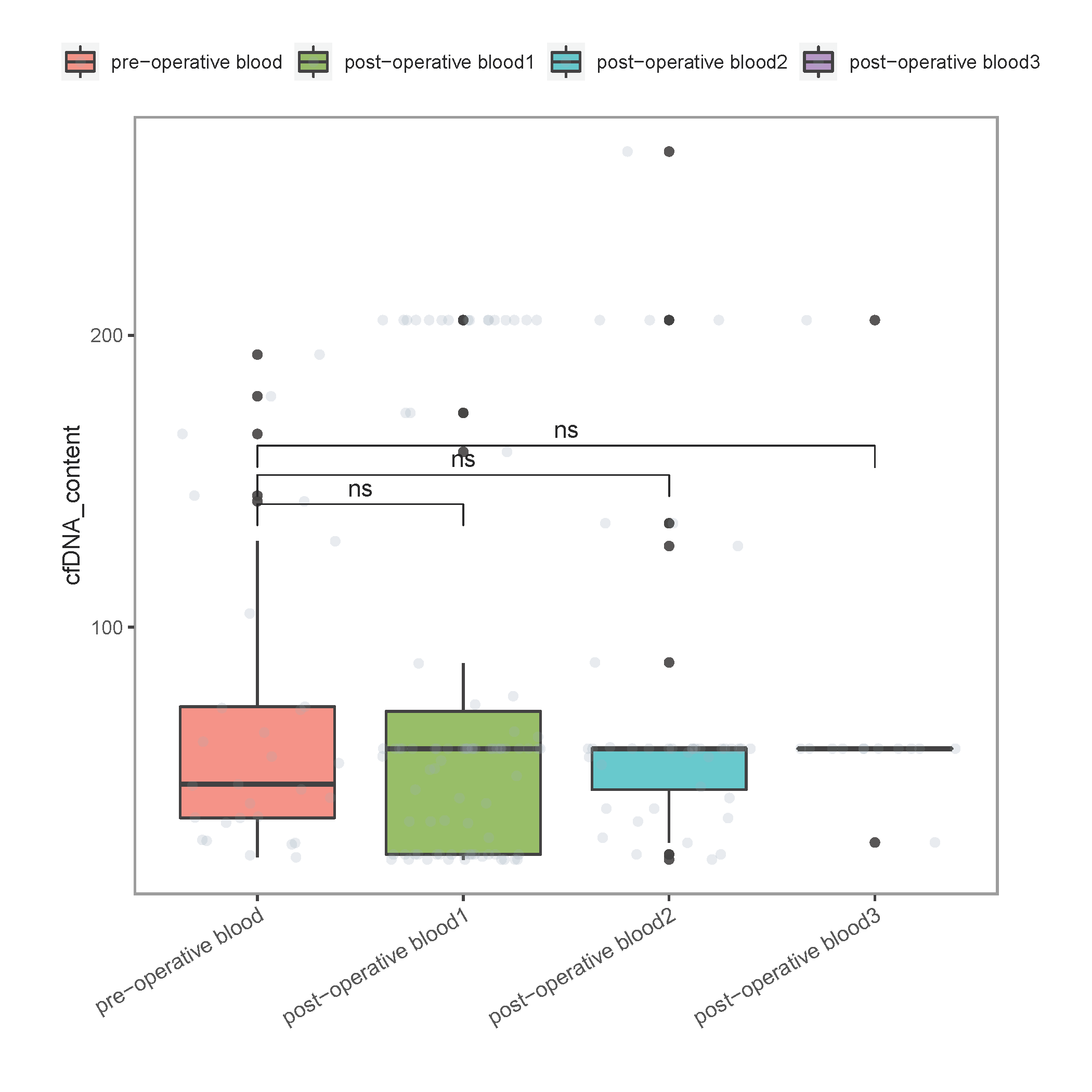

Supplement: Supplementary file 2 — Figure S1 [file CAM4-12-16687-s002.tiff]
