# Supplementary material for: Monitoring of gastrointestinal carcinoma via molecular residual disease with circulating tumor DNA using a tumor‐informed assay
Source: Cancer Med. 2023 Aug 21;12(16):16687–96. doi: 10.1002/cam4.6286 (PMC10501225; doi:10.1002/cam4.6286)
Supplement: Supplementary file 3 — Tables S1–S2 [file CAM4-12-16687-s001.docx]

**Table S1. The statistics of false positive sites detected in three negative samples.**

| **Number** | **Negative Sample** | **Chromosome** | **Locus** | **Reference Sequence** | **Variant Sequence** | **VAF** |
| --- | --- | --- | --- | --- | --- | --- |
| 1 | SNP1-N | chr3 | 150280445 | C | G | 0.02% |
| 2 | SNP1-N | chr1 | 158225019 | C | G | 0.06% |
| 3 | SNP1-N | chr4 | 90169925 | A | G | 0.06% |
| 4 | SNP1-N | chr19 | 1065197 | G | C | 0.05% |
| 5 | SNP2-N | chr9 | 25677698 | A | C | 0.02% |

**Table S2. List of results obtained from multiple monitored patients.**

| **Number** | **Tumor type** | **Stage** | **TNM Staging** | **Number of Detection Sites** | **Preoperative Blood** | **Postoperative Blood Sample 1** | **Postoperative Blood Sample 2** | **Postoperative Blood Sample 3** | **Postoperative Blood Sample 4** |
| --- | --- | --- | --- | --- | --- | --- | --- | --- | --- |
| 1 | Colorectal Adenocarcinoma | I | T2N0M0 | 36 | Negative | Negative | - | - | - |
| 2 | Colorectal Adenocarcinoma | I | T2N0M0 | 33 | Negative | Negative | Negative | - | - |
| 3 | Colorectal Adenocarcinoma | I | T2N0M0 | 38 | - | Negative | Negative | - | - |
| 4 | Colorectal Adenocarcinoma | I | T2N0M0 | 35 | - | Negative | Negative | Negative | - |
| 5 | Colorectal Adenocarcinoma | I | T2N0M0 | 32 | Positive | Negative | - | - | - |
| 6 | Colorectal Adenocarcinoma | I | T2N0M0 | 33 | - | Negative | Negative | - | - |
| 7 | Colorectal Adenocarcinoma | I | T2N0M0 | 31 | Negative | Negative | - | - | - |
| 8 | Colorectal Adenocarcinoma | I | T2N0M0 | 30 | - | Positive | Positive | Negative | Negative |
| 9 | Colorectal Adenocarcinoma | II | T3N0M0 | 33 | Positive | Negative | - | - | - |
| 10 | Colorectal Adenocarcinoma | II | T3N0M0 | 49 | Positive | Negative | - | - | - |
| 11 | Colorectal Adenocarcinoma | II | T3N0M0 | 31 | - | Negative | Negative | - | - |
| 12 | Colorectal Adenocarcinoma | II | T3N0M0 | 59 | Positive | Negative | - | - | - |
| 13 | Colorectal Adenocarcinoma | II | T3N0M0 | 44 | - | Negative | Negative | - | - |
| 14 | Colorectal Adenocarcinoma | II | T3N0M0 | 30 | Positive | Negative | - | - | - |
| 15 | Colorectal Adenocarcinoma | II | T3N0M0 | 42 | - | Negative | Negative | Negative | - |
| 16 | Colorectal Adenocarcinoma | II | T3N0M0 | 32 | Negative | Negative | - | - | - |
| 17 | Colorectal Adenocarcinoma | II | T3N0M0 | 30 | Positive | Negative | Negative | - | - |
| 18 | Colorectal Adenocarcinoma | III | T3N2M0 | 34 | Negative | Negative | - | - | - |
| 19 | Colorectal Adenocarcinoma | III | T3N2M0 | 40 | Positive | Positive | - | - | - |
| 20 | Colorectal Adenocarcinoma | III | T3N2M0 | 45 | - | Negative | Negative | - | - |
| 21 | Colorectal Adenocarcinoma | III | T4N1M0 | 39 | Positive | Negative | - | - | - |
| 22 | Colorectal Adenocarcinoma | III | T3N1M0 | 39 | Positive | Positive | - | - | - |
| 23 | Colorectal Adenocarcinoma | III | T3N1M0 | 35 | Positive | Positive | - | - | - |
| 24 | Colorectal Adenocarcinoma | III | T4N1M0 | 30 | - | Negative | Positive | - | - |
| 25 | Colorectal Adenocarcinoma | III | T3N1M0 | 35 | Positive | Negative | - | - | - |
| 26 | Colorectal Adenocarcinoma | III | T3N1M0 | 33 | - | Negative | Negative | - | - |
| 27 | Colorectal Adenocarcinoma | III | T3N1M0 | 36 | - | Negative | Negative | - | - |
| 28 | Colorectal Adenocarcinoma | III | T3N1M0 | 31 | - | Negative | Negative | - | - |
| 29 | Colorectal Adenocarcinoma | III | T3N1M0 | 39 | - | Negative | Negative | - | - |
| 30 | Colorectal Adenocarcinoma | III | T3N1M0 | 30 | - | Negative | Negative | Negative | - |
| 31 | Colorectal Adenocarcinoma | III | T3N1M0 | 33 | - | Positive | Negative | - | - |
| 32 | Colorectal Adenocarcinoma | III | T3N1M0 | 31 | - | Negative | Negative | Negative | - |
| 33 | Colorectal Adenocarcinoma | III | T2N1M0 | 30 | - | Negative | Negative | Negative | - |
| 34 | Colorectal Adenocarcinoma | III | T3N1M0 | 30 | - | Negative | Negative | Negative | - |
| 35 | Colorectal Adenocarcinoma | III | TxN1M0 | 33 | Positive | Negative | Negative | Negative | - |
| 36 | Colorectal Adenocarcinoma | III | T4N2M0 | 31 | Positive | Positive | - | - | - |
| 37 | Colorectal Adenocarcinoma | III | T3N1M0 | 30 | - | Negative | Negative | - | - |
| 38 | Colorectal Adenocarcinoma | IV | T4N2M1 | 43 | - | Positive | Positive | - | - |
| 39 | Colorectal Adenocarcinoma | IV | T2N0M1 | 48 | Positive | Positive | Positive | - | - |
| 40 | Colorectal Adenocarcinoma | IV | T4N1M1 | 32 | Positive | Negative | Negative | - | - |
| 41 | Colorectal Adenocarcinoma | IV | T3N1M1 | 39 |  | Negative | Negative | Negative | - |
| 42 | Colorectal Adenocarcinoma | IV | T3N2M1 | 30 | Negative | Positive | Negative | - | - |
| 43 | Colorectal Adenocarcinoma | IV | T3N1M1 | 30 | - | Positive | Negative | Negative | - |
| 44 | Colorectal Adenocarcinoma | IV | T4NxM1 | 30 | - | Positive | Negative |  | - |
| 45 | Colorectal Adenocarcinoma | IV | T4N1M1 | 55 | - | Negative | Negative | Negative | - |
| 46 | Colorectal Adenocarcinoma | - | - | 33 | - | Positive | Negative | Negative | - |
| 47 | Colorectal Adenocarcinoma | - | - | 30 | - | Positive | - | - | - |
| 48 | Gastric adenocarcinoma | III | T2N2M0 | 30 | Negative | Negative | - | - | - |
| 49 | Gastric adenocarcinoma | IV | T3N1M1 | 30 | - | Negative | Negative | - | - |
| 50 | Gastric adenocarcinoma | I | T2N0M0 | 38 | Positive | Negative | Negative | - | - |
| 51 | Gastric adenocarcinoma | III | T3N3M0 | 30 | - | Negative | Positive | - | - |
| 52 | Gastric adenocarcinoma | III | T3N3M0 | 20 | - | Negative | Positive | - | - |
| 53 | Gastric adenocarcinoma | II | T3N1M0 | 30 | - | Positive | Positive | - | - |
| 54 | Gastric adenocarcinoma | III | T4aN2M0 | 30 | - | Negative | Negative | - | - |
| 55 | Gastric adenocarcinoma | III | T2N3M0 | 34 | - | Positive | Positive | - | - |
| 56 | Small intestinal stromal tumor | - | - | 22 | Negative | Negative | - | - | - |
| 57 | Small intestinal stromal tumor | - | - | 30 | - | Positive | Negative | _ | - |
| 58 | Adenocarcinoma of small intestine | - | - | 30 | - | Negative | Negative | Negative | - |
| 59 | Duodenal malignant tumors | IV | - | 30 | - | Negative | Negative | - | - |
